# Risk Factors of External Ventricular Drainage-Related Infections: A Retrospective Study of 147 Pediatric Post-tumor Resection Patients in a Single Center

**DOI:** 10.3389/fneur.2019.01243

**Published:** 2019-11-28

**Authors:** Pengwei Lu, Raynald, Wei Liu, Jian Gong, Tao Sun, Chunde Li, Lukman Ma′ruf, Yanzhu Fan, Ruifang Zhu, Yongji Tian

**Affiliations:** ^1^Department of Neurosurgery, Beijing Tiantan Hospital, Capital Medical University, Beijing, China; ^2^Department of Interventional Neurology, Beijing Tiantan Hospital, Capital Medical University, Beijing, China; ^3^Department of Neurosurgery, Gatot Subroto Central Army Hospital, Jakarta, Indonesia

**Keywords:** EVD, tumor surgery, risk factors, infection, children

## Abstract

**Background:** External ventricular drainage (EVD) is common after brain surgery. However, the incidence of EVD-related infection (ERI) is still relatively high and can increase morbidity and mortality.

**Objective:** The objective of this study was to analyze ERI factors in pediatric population post-brain tumor surgery.

**Methods:** From January 2016 to December 2017, 147 patients <18 years old underwent tumor removal at Beijing Tiantan Hospital and had postoperative EVD. We recorded basic demographic data as well as several risk factors. We then analyzed whether these factors were related to ERI.

**Results:** Patients with a preoperative ventriculo-peritoneal (V-P) shunt, those with longer operation time, those who received blood transfusion, those with more frequent cerebrospinal fluid (CSF) sampling, and those with longer indwelling time of EVD had higher risks of infection (*p* < 0.05). Logistic regression analysis confirmed that a preoperative V-P shunt, operative duration, intraoperative blood transfusion, frequency of CSF sampling, and EVD duration were correlated with postoperative ERI (*p* < 0.05).

**Conclusion:** EVD should be removed as soon as possible and any unnecessary procedures should be avoided to reduce the infection rate. However, prophylactic treatment should be given in case patients do not meet the indication for EVD removal.

## Introduction

External ventricular drainage (EVD) is a common practice after brain tumor surgery since it helps monitor intracranial pressure and drain the residual blood (1–4). Unfortunately, EVD is also a potential access point for organisms to enter the intracranial compartment. The incidence of EVD-related infection (ERI) may reach up to 22% in high-risk patients (5, 6). ERI increases morbidity and mortality as well as prolonging hospital stays, increasing hospitalization costs, and even leading to multiple surgeries (7, 8). Therefore, ERI has attracted great attention from experts, and a recent meta-analysis study revealed several risk factors such as duration of EVD monitoring, systemic infection, presence of intraventricular hemorrhage, basilar skull fractures with a CSF leak, catheter manipulations, and leakage around the EVD catheter. However, there are few reports regarding intracranial infection in children with EVD, especially after brain tumor surgery. The purpose of this study was to investigate and analyze several risk factors of ERI in post-brain tumor surgery to elucidate the best treatment strategy to achieve a good prognosis.

## Patients and Methods

We retrospectively studied 147 patients <18 years old from January 2016 to December 2017 with a confirmed diagnosis of intracranial infection after tumor resection. This study was approved by the Ethics Committee of Beijing Tiantan Hospital, Capital Medical University. Informed consent was obtained from all participants or their parents or legal guardians.

**Figure 1:**
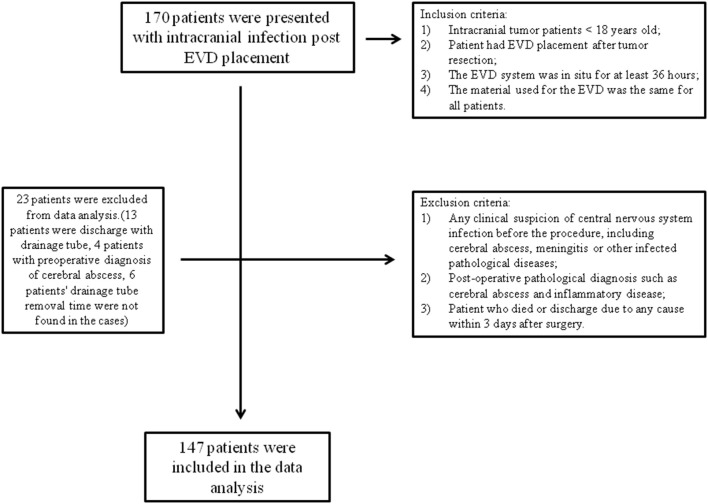
Flow chart of the patients included in this study.

Our inclusion criteria were as follows: (1) intracranial tumor patients who were <18 years old; (2) the patient had EVD placement after tumor resection; (3) the EVD system was *in situ* for at least 36 h; and (4) the material used for the EVD was the same for all patients. Exclusion criteria were (1) any clinical suspicion of central nervous system infection before the procedure, including cerebral abscess, meningitis, or other infectious pathological diseases; (2) a postoperative pathological diagnosis such as a cerebral abscess or inflammatory disease; and (3) a patient who died (due to progression of the disease or severe postoperative complications) or was discharged due to any cause within 3 days after surgery, such as a financial problem or the patient's family of their own free will requesting discharge from the hospital (Figure 1).

### ERI Diagnosis

Criteria for diagnosis of ERI were as follows: (1) clinical signs such as fever, altered consciousness, nuchal rigidity, focal neurologic deficits, and positive meningitis sign (e.g., Kernig sign); (2) cerebrospinal fluid (CSF) glucose level <2.5 mmol/L, leukocytes >100/μl; (3) postoperative peripheral blood WBC > 10,000/μl, Neu% >75%. Patients meeting criteria 1 and 2 or 3 were diagnosed as having ERI. All CSF samples were obtained from the EVD system.

### Data Collection

We recorded basic demographic data regarding age, sex, and place of origin, as well as several risk factors such as preoperative inpatient duration, operative duration (<4 h; ≥4 h), length of stay in hospital, course of the disease, presence of a ventriculo-peritoneal (V-P) shunt before tumor resection surgery, tumor volume (<4 cm; ≥4 cm), extent of tumor resection, and past history of intracranial surgery, intraoperative blood transfusion (0; 0–400 ml; >400 ml). We also collected data on duration of EVD stay and the total number of times CSF was sampled prior to diagnosis of the infection. In addition, we also collected information regarding the tumor location, characteristics, and outcome of the treatment.

### Statistics

Statistical analyses were performed using SPSS software 23.0. All measured data were calculated by mean ± standard deviation by ($\bar{x}$ ± s). For the normal distribution and equal variances data, the statistical analyses of categorical variables between two groups were carried out using *t*-tests. For abnormal distributions and unequal variances data, the statistical analyses of categorical variables between the two groups were carried out using non-parametric tests (Mann–Whitney *U*-test, Kruskal–Wallis *H-*test) as appropriate. Correlation of risk factors and infection were evaluated by binary logistic regression. Statistical significance was defined as a *P*-value < 0.05.

## Results

### Patient Demographic Data

**Figure 2:**
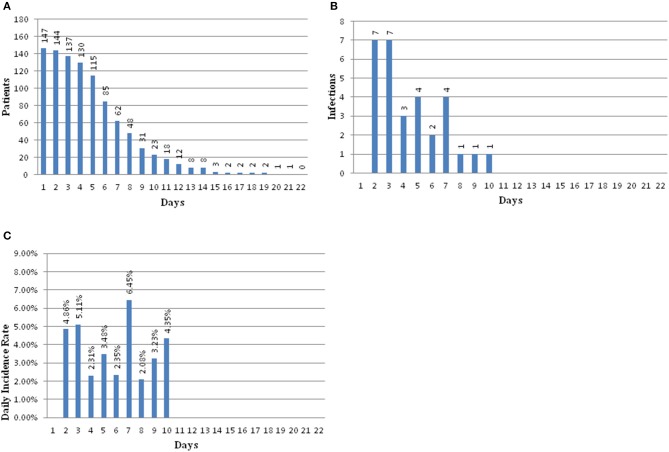
**(A)** Bar graph showing patients at risk for infection over time. **(B)** Bar graph showing number of infections over time. **(C)** Bar graph showing the daily infection rate over time.

There were 147 pediatric patients who received EVD after tumor resection surgery that were included in this study. Among them, 30 had intracranial infection after operation, so the incidence was 20.4%. The patients were divided into an infection group and a non-infection group. The average hospitalization days of the infected group and non-infected group were 23.67 (±9.58) days and 15.51 (±5.09) days, respectively. There was a significant difference between the two groups (*t* = 11.51, *p* = 0.001) (Table 1). The infection occurred during the 10 days after EVD placement. Early infection occurred on the second day after EVD placement with an incidence of 4.86%, 5.11% on the third day, 2.31% on the fourth day, 3.48% on the fifth day, 2.35% on the sixth day, 6.45% on the seventh day, 2.08% on the eighth day, and 3.23% on the ninth day. The latest period of infection occurred on the 10th day after EVD placement with an incidence of 4.35%. There were 18 patients who had EVD placement for longer than 10 days; however, no infections were found in these patients (Figure 2).

**Table 1:** Characteristics of the patients.

**Variables**	**Mean** **±** **SD**		**P50 (P25, P75)**	
Age	7.15 ± 3.91			
Pre-operative inpatient (d)	4.76 ± 2.49			
Operative duration (h)	3.79 ± 1.55			
Duration use of EVD (d)	7.67 ± 3.31			
Inpatient (d)	17.18 ± 7.04			
Course of disease (d)			30 (15, 90)	
**Variables**	**Infection group** **(Mean** **±** **SD)**	**Noninfection group** **(Mean** **±** **SD)**	***t***	***p***
Age	7.00 ± 4.10	7.19 ± 3.88	1.116	0.292
Pre-op inpatient (d)	5.50 ± 2.90	4.56 ± 2.35	3.824	0.052
Operative duration (h)	4.66 ± 2.00	3.57 ± 1.33	6.159	**0.014**
Duration of EVD (d)	10.00 ± 4.44	7.08 ± 2.66	4.609	**0.000**
Inpatient (d)	23.67 ± 9.58	15.51 ± 5.09	11.512	**0.001**

*Boldface type indicates statistical significance*.

The average age of the children was 7.15 ± 3.91 years old. The average length of hospitalization before the operation was 4.76 ± 2.49 days. The average length of the operation was 3.79 ± 1.55 h, and the average time of EVD was 7.67 ± 3.31 days. The average total duration of hospitalization was 17.18 ± 7.04 days. The median duration was 30 days (Table 1). There was male predominance with a male-to-female ratio of 1.6:1. Of all 147 patients, 91 were male (61.9%) and 56 were female (38.1%). Seventy-four (50.3%) patients were from cities, and 63 (49.7%) patients were from rural area. Of the 147 patients, 30 patients had a V-P shunt (20.4%) prior to tumor resection surgery. Seventy-nine (53.7%) patients had tumors larger than or equal to 4 cm in diameter, and 68 (46.3%) patients had a tumor <4 cm in diameter. Total resection of the tumors was achieved in 108 (73.5%) of the patients as confirmed by postoperative MRI. Sixteen (10.9%) patients had a history of craniotomy. Forty-five (30.6%) patients had a blood transfusion during the operation. One hundred four (70.7%) patients had CSF samples collected fewer than three times a week after the operation (Table 2).

**Table 2:** Risk factors related to ERI.

**Variables**	**No. patient**	**Infection group**	**Noninfection group**	**χ2**	***P***
Total	147	30	117		
**Age**					
≤7 year	81 (55.1)	15 (18.5)	66 (81.5)	0.397	0.529
>7 year	66 (44.9)	15 (22.7)	51 (77.3)		
**Sex**					
Male	91 (61.9)	16 (17.6)	75 (82.4)	1.174	0.279
Female	56 (38.1)	14 (25.0)	42 (75.0)		
**Native place**					
City	74 (50.3)	16 (21.6)	58 (78.4)	0.135	0.713
Country	73 (49.7)	14 (19.2)	59 (80.8)		
**Pre-op VP shunt**					
Yes	30 (20.4)	12 (40.0)	18 (60.0)	8.907	**0.003**
No	117 (79.6)	18 (15.4)	99 (84.6)		
**Operative duration**					
<4 h	68 (46.3)	9 (13.2)	59 (86.8)	4.008	**0.045**
≥4 h	79(53.7)	21 (26.6)	58 (73.4)		
**Tumor diameter**					
<4 cm	68 (46.3)	18 (26.5)	50 (73.5)	2.863	0.091
≥4 cm	79 (53.7)	12 (15.2)	67 (84.8)		
**Post-op tumor residue**					
Yes	39 (26.5)	7 (17.9)	32 (82.1)	0.198	0.657
No	108 (73.5)	23 (21.3)	85 (78.7)		
**Past history of craniotomy**					
Yes	16 (10.9)	1 (6.3)	15 (93.7)	1.346	0.246
No	131 (89.1)	29 (22.1)	102 (77.9)		
**Blood transfusion**					
Yes	45 (30.6)	16 (35.6)	29 (64.4)	9.161	**0.002**
No	102 (69.4)	14 (13.7)	88 (86.3)		
**Blood transfusion volume**					
0	102 (69.4)	14 (13.7)	88 (86.3)	17.438	**0.000**
0–400	30 (20.4)	7 (23.3)	23 (76.7)		
>400	15 (10.2)	9 (60.0)	6 (40.0)		
**CSF collection**					
<3 times/week	104 (70.7)	7 (6.7)	97 (93.3)	40.946	**0.000**
≥3 times/week	43 (29.3)	23 (53.5)	20 (46.5)		
**EVD duration**					
>7 days	62 (42.2)	19 (30.6)	43 (69.4)	6.918	**0.009**
≤7 days	85 (57.8)	11 (12.9)	74 (87.1)		

*Boldface type indicates statistical significance*.

### Factors Associated With ERI

Patients were divided into the infection group and non-infection group according to the postoperative incidence of intracranial infection. The clinical data pre-, intra-, and post-operation were compared and observed, including the patient's age, sex, days of hospitalization before the operation, place of origin, preoperative V-P shunt, duration of operation (<4 h; ≥4 h), tumor volume (≥4 cm; <4 cm), extent of tumor resection (tumor residue), history of craniotomy, intraoperative blood transfusion (0; 0–400 ml; >400 ml), sampling frequency of CSF (less than three times a week; more than three times a week), and time of EVD (≤7; >7 days). In this study, the operation time of the infection group was longer than that of the non-infection group (4.66 ± 2.00 vs. 3.57 ± 1.33; *t* = 6.159, *p* = 0.014). Moreover, the duration for EVD of the infection group was longer than that in the non-infection group (10.00 ± 4.44 vs. 7.08 ± 2.66; *t* = 4.609, *p* = 0.000). The infection rate of children with a preoperative V-P shunt was higher than that of children without a V-P shunt (χ^2^ = 8.907, *p* = 0.003). The infection rate of children with a longer operation duration (≥4 h) was higher than that of children with shorter operation duration (<4 h) (χ^2^ = 4.008, *p* = 0.045). The infection rate of children with an intraoperative transfusion was 35.6%, which is higher than that of children without transfusion (χ^2^ = 9.161, *p* = 0.002). The blood transfusion volume was set at 400 ml as the cutoff (0, 0–400 ml, and >400 ml). The higher the volume of the transfusion was, the higher the infection rate (χ^2^ = 17.438, *p* = 0.000). The infection rate of the group whose CSF sampling frequency was more than three times a week was higher than that of the group with sampling fewer than three times a week (χ^2^ = 40.946, *p* = 0.000). In addition, the time of drainage tube placement was classified as a categorical variable. The infection rate increased significantly as the drainage tube was placed for a longer duration 12.9% (≤7 days group) vs. 30.6% (>7 days group) (χ^2^ = 6.918, *p* = 0.009) (Table 2).

### Tumor Characteristics and Outcomes

In 30 patients who had ERI, the tumors were located in the posterior third ventricle/pineal region (20%), cerebral hemisphere (16.7%), sellar region (16.7%), posterior fossa (16.7%), lateral ventricle (13.3%), third ventricle (10%), thalamus (3.3%), and basal ganglia (3.3%). Their pathological findings were astrocytoma (16.7%), ependymoma (13.3%), germ cell tumor (13.3%), medulloblastoma (10%), germinoma (6.7%), teratoma (mature/immature) (6.7%), craniopharyngioma (6.7%), choroid plexus papilloma (6.7%), meningioma (6.7%), pinealocytoma (3.3%), subependymal giant cell astrocytoma (3.3%), hemangioblastoma (3.3%), and atypical rhabdoid tumor (3.3%). Of 30 ERI patients, gross total resection (GTR) was achieved in 80% of the cases, and subtotal resection was achieved in 20% of the cases (Supplementary Tables 1, 2). There were no significant differences for the ratio of the infectious group and noninfectious group in regard to different tumor locations (Supplementary Table 3).

Thirty patients who had ERI underwent CSF culture, and only 5 of 30 cases (16.7%) were found to have a positive CSF culture. The organisms found in these cultures were *Staphylococcus* in two patients, *Pseudomonas aeruginosa* in two patients, and Acinetobacter in one patient. The patient's outcomes were assessed at the time of discharge with the Glasgow Outcome scale (GOS), which ranges from 1 to 5. A score of 5 indicates mild or no disability, while an adverse clinical outcome was defined as a score of 1 to 4. In the infection group, 10 patients had a GOS of 5 and 20 patients had a GOS <5, while in the non-infection group 26 patients had a GOS of 5, while 91 patients had a GOS <5. Although most of the patients had an adverse clinical outcome (GOS < 5), there was no significant difference between groups (Supplementary Table 4).

### Univariate and Multivariate Analysis

According to the univariate analysis (Table 3), the duration of the operation, the time of drainage tube placement, whether there was a V-P shunt before the operation, a blood transfusion during the operation, and sampling frequency of CSF were set as independent variables, and whether infection occurred was set as the dependent variable. Logistic regression analysis showed that preoperative V-P shunt (OR: 3.667, 95% CI: 1.511–8.897, *p* = 0.004), operative duration (OR: 0.421, 95% CI: 0.178–0.996, *p* = 0.049), intraoperative blood transfusion (OR: 3.468, 95% CI: 1.511–7.961, *p* = 0.003), sampling frequency of CSF (OR: 0.556, 95% CI: 0.449–0.689, *p* = 0.000), and EVD duration (OR: 0.774, 95% CI: 0.678–0.883, *p* = 0.000) were correlated with postoperative ERI.

**Table 3:** Univariate analysis.

**Variables**	**HR**	**CI 95%**	***P***
**Age**			
≤7 year	1	1	
>7 year	0.773	0.346–1.726	0.529
**Sex**			
Male	1	1	
Female	0.640	0.285–1.440	0.281
**Native place**			
City	1	1	
Country	1.163	0.520–2.597	0.713
**Pre-op VP shunt**			
Yes	1	1	
No	3.667	1.511–8.897	**0.004**
**Operative duration**			
<4 h	1	1	
≥4 h	0.421	0.178–0.996	**0.049**
**Tumor diameter**			
<4 cm	1	1	
≥4 cm	2.010	0.888–4.551	0.094
**Post-op tumor residue**			
Yes	1	1	
No	1.237	0.484–3.162	0.657
**Past history of craniotomy**			
Yes	1	1	
No	0.234	0.030–1.851	0.169
**Blood transfusion**			
Yes	1	1	
No	3.468	1.511–7.961	**0.003**
**CSF collection**			
<3 times/week	1	1	
≥3 times/week	0.556	0.449–0.689	**0.000**
**EVD duration**			
>7 days	1	1	
≤7 days	0.774	0.678–0.883	**0.000**

*Boldface type indicates statistical significance*.

According to the multivariate analysis (Table 4), operative duration (OR: 0.092, 95% CI 0.017–0.506, *p* = 0.006), sampling frequency of CSF (OR: 0.024, 95% CI: 0.005–0.122, *p* = 0.000), and EVD duration (OR: 0.749, 95% CI: 0.602–0.933, *p* = 0.010) had a strong correlation with postoperative ERI, while age, sex, native place of origin, preoperative V-P shunt, tumor diameter, postoperative tumor residue, past history of craniotomy, and blood transfusion did not show a correlation with ERI.

**Table 4:** Multivariate analysis.

**Variables**	**HR**	**CI 95%**	***P***
**Age**			
≤7 year	1	1	
>7 year	0.666	0.150–2.953	0.593
**Sex**			
Male	1	1	
Female	0.362	0.084–1.552	0.171
**Native place**			
City	1	1	
Country	2.167	0.520–9.029	0.288
**Pre-op VP shunt**			
Yes	1	1	
No	4.213	0.828–21.437	0.083
**Operative duration**			
<4 h	1	1	
≥4 h	0.092	0.017–0.506	**0.006**
**Tumor diameter**			
<4 cm	1	1	
≥4 cm	4.444	0.869–22.732	0.073
**Post-op tumor residue**			
Yes	1	1	
No	3.192	0.579–17.605	0.183
**Past history of craniotomy**			
Yes	1	1	
No	0.140	0.008–2.423	0.177
**Blood transfusion**			
Yes	1	1	
No	2.341	0.528–10.378	0.263
**CSF collection**			
<3 times/week	1	1	
≥3 times/week	0.024	0.005–0.122	**0.000**
**EVD duration**			
>7 days	1	1	
≤7 days	0.749	0.602–0.933	**0.010**

*Boldface type indicates statistical significance*.

## Discussion

Previous studies have identified numerous risk factors of intracranial infections related to EVD placement, including multiple insertions, CSF leak, surgical intervention, systemic infection, longer operative time, failing to maintain a closed ventricular system, non-sterile technique during the procedure, delayed replacement of an EVD that became compromised, and delayed removal of the EVD even when clinically indicated (9–15). However, there is still no study that has focused on the incidence and risk factors of EVD related to intracranial infection post-brain tumor surgery in a pediatric population. Considering that intracranial infection can cause a devastating result, especially in a pediatric population that has a relatively weak immune system relative to the adult population, special attention and precautions are needed. Therefore, we retrospectively studied and analyzed 147 pediatric patients from our institution that had EVD post-brain tumor surgery.

The youngest patient in this study was 8 months, and the oldest was 18 years. The average age was 7.15 ± 3.91 years old. There was a male predominance accounting for 61.9% of the patients, and the male-to-female ratio was 1.6:1. There were no significant differences in the infection rate in different age populations, sex, place of origin, tumor diameter, postoperative tumor residue, or past history of craniotomy. However, there was a significant difference in the infection rate with regard to preoperative V-P shunt, operation duration, blood transfusion, CSF collection, and EVD duration among different populations. This result suggests that the incidence of infection is related more to the hospital than to factors associated with the patient such as demographic and origin.

Operative duration is an independent factor for ERI. In our study, the infection rate was 26.6% in the patient group who had an operation time ≥4 h and 13.2% in the patient group with an operation time <4 h (χ^2^ = 4.008, *p* = 0.045). This result suggests that a prolonged duration of the operation can significantly increase the chance of a contaminated surgical site infection due to longer exposure time of the brain tissue to the surrounding air. Routine CSF sampling is usually performed two to three times and if clinical symptoms of ERIs occur, repeated sampling can be performed. Our results showed that CSF sampling frequency was also an independent factor for ERIs with an infection rate of 53.5% in CSF sampling frequency ≥3 times per week and 6.7% in <3 times per week (χ^2^ = 40.946, *p* = 0.000). This result is consistent with Hoefnagel et al. (16). The higher the sampling frequency is, the higher the infection rate. Therefore, we suggest that conventional CSF sampling is not recommended without the presence of significant clinical signs.

A preoperative V-P shunt is considered to be a risk factor for ERI in our study although it is not an independent factor (χ^2^ = 8.907, *p* = 0.003). The infection rate of children with a preoperative V-P shunt was 40%, which is higher than that of children without a preoperative V-P shunt (15.4%). The incidence of intracranial infection after V-P shunt placement in neurosurgical practice is relatively high. This incidence may be attributed to inadequate preoperative preparation, inappropriate timing of operation, lack of standardization of aseptic operation, or longer exposure duration during the procedure.

Massive bleeding is sometimes unavoidable during tumor resection, especially of tumors with invasive characteristics or those located adjacent to vascular structures. Often colloid and/or crystalline fluid and blood is needed to maintain the stability of blood circulation. However, blood transfusion can lead to an imbalance in the proportion of white blood cells in patients and interfere in the functions of immune cells, which can lead to the patient being more vulnerable to infection. This mechanism may explain why the infection rate of children with intraoperative transfusion was higher (35.6%) than that of children without transfusion (13.7%), and the higher the transfusion volume is, the higher the risk of infection (χ^2^ = 17.438, *p* = 0.000).

The indwelling time of a ventricular drainage tube is considered to be the most important independent risk factor, although there is no consensus among experts. A study by Park et al. (17) showed that the infection rate increased during the 4 days post-catheterization, and then became stable afterwards. The infection rate did not increase even if the catheterization time exceeded 10 days, with an average infection time of 8.6 days. Strojnik et al. (18) reported that the average indwelling time of an EVD tube was 7.41 ± 3.26 days, and he considered that day 7 is the crucial time for the indwelling of EVD. He reported that the infection rate at <7 days was 9.46% and that at >7 days, it was 23.44%. In 2014, a large sample survey of 755 EVD patients was performed by Huashan Hospital, and they found that the infection rate of 61 EVD patients increased rapidly from the 5th to the 12th day, and then stabilized after the 12th day. The average indwelling time of the infected patients was 10.06 days, while the average indwelling time of the non-infected patients was 5.53 days (19). Consistent with Strojnik et al., in our study, the infection rate was 12.9% when the indwelling time of EVD was <7 days and 30.6% when the indwelling time of the EVD tube was more than 7 days. From these results, there should be a consensus agreement regarding the indwelling time for EVD. We suggest that EVD should be removed as soon as possible for patients who meet the indication of EVD removal. During the procedure, strict aseptic operation, avoidance of leakage and reflux of the drainage tube, prevention of liquid contact between the outlet of the drainage tube and the CSF collection bottle, and clamping the drainage tube should be conducted for prevention of intracranial infection. However, in the case of a patient who does not meet the indication for EVD removal, early application of broad-spectrum antibiotics can be considered for prophylaction (20).

In our study, we did not find any correlation between tumor location and the incidence of ERI. Unfortunately, due to a lack of samples to study, we did not further evaluate the correlation between tumor histology and the incidence of ERI. In our study, only 5 of 30 patients who had ERI had a positive CSF culture, and prior use of antibiotics may have caused the increased false-negative results for CSF analysis (21, 22). There was no significant difference between patient GOS in the infection group and the non-infection group. However, further studies with more patients are needed for verification.

The major strength of our analysis is that it is the first to assess and discuss the ERI factors in a pediatric population post-brain tumor surgery, which we believe has distinct characteristics from adult populations or other neurosurgical circumstances. However, the lack of samples to study might not have provided an accurate reflection of the results, and several risk factors such as preoperative V-P shunt and intraoperative blood transfusion are still ambiguous and might cause confusion between postoperative infection and ERI. Therefore, more integrative and comprehensive evaluation of patient data with strict screening is needed in future studies, and moreover, a comparison with an adult population and other neurosurgical circumstances should be performed to validate the results.

## Conclusion

A preoperative V-P shunt, operative duration, intraoperative blood transfusion, CSF sampling frequency, and indwelling time of the ventricular drainage tube are all important factors that affect the incidence of ERI. ERI can be fatal to patients, and thus, intensive care is important to reduce its morbidity and mortality. In the case of a patient who does not meet the indication for EVD removal, early prophylaxis and treatment is mandatory.

## Data Availability Statement

All datasets generated for this study are included in the article/Supplementary Material.

## Ethics Statement

The studies involving human participants were reviewed and approved by This study was approved by the Ethics Committee of Beijing Tiantan Hospital, Capital Medical University. Written informed consent to participate in this study was provided by the participants' legal guardian/next of kin.

## Author Contributions

YT conceived and led the project. PL, YF, and RZ performed data collection and analysis. R, WL, JG, TS, CL, and LM performed quality control of the data. PL and R co-wrote the manuscript with input from all co-authors.

### Conflict of Interest

The authors declare that the research was conducted in the absence of any commercial or financial relationships that could be construed as a potential conflict of interest.
