# Supplementary material for: Risk Factors of External Ventricular Drainage-Related Infections: A Retrospective Study of 147 Pediatric Post-tumor Resection Patients in a Single Center
Source: Front Neurol. 2019 Nov 28;10:1243. doi: 10.3389/fneur.2019.01243 (PMC6892983; doi:10.3389/fneur.2019.01243)
Supplement: Supplementary file 1 [file Table_2.docx]

**Supplementary Table 1. Characteristics of the tumor, type of infection and outcome of the infectious group**

| **No** | **Tumor location** | **Pathology** | **Resection degree** | **Bacteria** | **Type of infection** | **Outcome** |
| --- | --- | --- | --- | --- | --- | --- |
| 1 | Posterior fossa | Ependymoma | GTR | Staphylococcus aureus | Bacterial | Improve |
| 2 | Thalamus | Atypical rhabdoid tumor | GTR | Negative CSF culture | No | Cure |
| 3 | Cerebral hemisphere | Ependymoma | GTR | Negative CSF culture | No | Cure |
| 4 | Ventricle | Ependymoma | GTR | Negative CSF culture | No | Cure |
| 5 | Ventricle | Subependymal giant cell astrocytoma | GTR | Negative CSF culture | No | Improve |
| 6 | Pineal | Mixed germ cell tumor | GTR | Negative CSF culture | No | Improve |
| 7 | Ventricle | Germ cell tumor | Subtotal | Negative CSF culture | No | Improve |
| 8 | Pineal | Pinealocytoma | GTR | Negative CSF culture | No | Cure |
| 9 | Basal ganglia | Germinoma | GTR | Negative CSF culture | No | Improve |
| 10 | Posterior 3rd ventricle | Teratoma | GTR | Pseudomonas aeroginosa | Bacterial | Improve |
| 11 | Posterior fossa | Astrocytoma | GTR | Negative CSF culture | No | Cure |
| 12 | Ventricle | Choroid plexus papilloma | GTR | Negative CSF culture | No | Improve |
| 13 | Sellar area | Astrocytoma | Subtotal | Staphylococcus aureus | Bacterial | Improve |
| 14 | 3rd Ventricle | Choroid plexus papilloma | GTR | Negative CSF culture | No | Cure |
| 15 | Cerebral hemisphere | Ependymoma | GTR | Negative CSF culture | No | Improve |
| 16 | 3rd Ventricle | Astrocytoma | GTR | Negative CSF culture | No | Cure |
| 17 | Posterior fossa | Medulloblastoma | GTR | Negative CSF culture | No | Cure |
| 18 | 3rd Ventricle | Mixed germ cell tumor | Subtotal | Negative CSF culture | No | Cure |
| 19 | Posterior fossa | Medulloblastoma | GTR | Acinetobacter | Bacterial | Improve |
| 20 | Sellar area | Astrocytoma | Subtotal | Negative CSF culture | No | Improve |
| 21 | Sellar area | Craniopharyngioma | GTR | Pseudomonas aeroginosa | Bacterial | Cure |
| 22 | Sellar area | Astrocytoma | Subtotal | Negative CSF culture | No | Improve |
| 23 | Sellar area | Craniopharyngioma | GTR | Negative CSF culture | No | Cure |
| 24 | Posterior 3rd ventricle | Germinoma | GTR | Negative CSF culture | No | Improve |
| 25 | Posterior 3rd ventricle | Mature teratoma | GTR | Negative CSF culture | No | Cure |
| 26 | Cerebral hemisphere | Meningioma | GTR | Negative CSF culture | No | Cure |
| 27 | Posterior fossa | Hemangioblastoma | GTR | Negative CSF culture | No | Improve |
| 28 | Posterior fossa | Medulloblastoma | GTR | Negative CSF culture | No | Improve |
| 29 | Cerebral hemisphere | Meningioma | Subtotal | Negative CSF culture | No | Improve |
| 30 | Cerebral hemisphere | Germ cell tumor | GTR | Negative CSF culture | No | Improve |

GTR: Gross total resection; CSF: Cerebrospinal fluid

**Supplementary Table 2. Data of the tumor characteristics and CSF culture in the infectious group**

| **Variable** | N | % |
| --- | --- | --- |
| Tumor location |  |  |
| Posterior 3rd ventricle / pineal | 6 | 20 |
| Cerebral hemisphere | 5 | 16.7 |
| Sellar | 5 | 16.7 |
| Posterior fossa | 5 | 16.7 |
| Lateral ventricle | 4 | 13.3 |
| 3rd ventricle | 3 | 10 |
| Thalamus | 1 | 3.3 |
| Basal ganglia | 1 | 3.3 |
| Pathology |  |  |
| Astrocytoma | 5 | 16.7 |
| Ependymoma | 4 | 13.3 |
| Germ cell tumor | 4 | 13.3 |
| Medulloblastoma | 3 | 10 |
| Germinoma | 2 | 6.7 |
| Teratoma (mature/immature) | 2 | 6.7 |
| Craniopharyngioma | 2 | 6.7 |
| Choroid plexus papilloma | 2 | 6.7 |
| Meningioma | 2 | 6.7 |
| Pinealocytoma | 1 | 3.3 |
| Subependymal giant cell astrocytoma | 1 | 3.3 |
| Hemangioblastoma | 1 | 3.3 |
| Atypical rhabdoid tumor | 1 | 3.3 |
| Resection degree |  |  |
| GTR | 24 | 80% |
| Subtotal | 6 | 20% |
| CSF culture |  |  |
| Positive | 5 | 16.7 |
| Negative | 25 | 83.3 |

**Supplementary Table 3. Relation of the tumor location and the EVD-related infections**

| **Tumor location** | **Infectious group** | **Non-infectious group** | **χ2** | **P** |
| --- | --- | --- | --- | --- |
| Posterior fossa | 5 | 19 | 3.430 | 0.634 |
| Post 3rd ventricle / pineal | 6 | 27 |  |  |
| Thalamus / basal ganglia | 2 | 17 |  |  |
| Sellar area | 5 | 18 |  |  |
| Ventricle | 7 | 14 |  |  |
| Cerebral hemisphere | 5 | 22 |  |  |

**Supplementary Table 4 GOS of the infection and non infection group**

| **GOS** | **Infectious group** | **Non-infectious group** | **χ2** | **P** |
| --- | --- | --- | --- | --- |
| 2 | 0 | 4 | 3.192 | 0.363 |
| 3 | 10 | 41 |  |  |
| 4 | 10 | 46 |  |  |
| 5 | 10 | 26 |  |  |

| **GOS** | **Infectious group** | **Non-infectious group** | **χ2** | **P** |
| --- | --- | --- | --- | --- |
| 5 | 10 | 26 | 3.354 | 0.067 |
| < 4 | 20 | 91 |  |  |
